# Prognostic value of tumor suppressors in osteosarcoma before and after neoadjuvant chemotherapy

**DOI:** 10.1186/s12885-015-1397-4

**Published:** 2015-05-09

**Authors:** Bernhard Robl, Chantal Pauli, Sander Martijn Botter, Beata Bode-Lesniewska, Bruno Fuchs

**Affiliations:** 1 Laboratory for Orthopedic Research, Department of Orthopedics, Balgrist University Hospital, Forchstrasse 340, 8008 Zurich, Switzerland; 2 Institute of Surgical Pathology, University Hospital Zurich, Zurich, Switzerland

**Keywords:** P53, PTEN, P16, Osteosarcoma, Chemotherapy, Tissue Microarray, Tumor Suppressor Genes

## Abstract

**Background:**

Primary bone cancers are among the deadliest cancer types in adolescents, with osteosarcomas being the most prevalent form. Osteosarcomas are commonly treated with multi-drug neoadjuvant chemotherapy and therapy success as well as patient survival is affected by the presence of tumor suppressors. In order to assess the prognostic value of tumor-suppressive biomarkers, primary osteosarcoma tissues were analyzed prior to and after neoadjuvant chemotherapy.

**Methods:**

We constructed a tissue microarray from high grade osteosarcoma samples, consisting of 48 chemotherapy naïve biopsies (BXs) and 47 tumor resections (RXs) after neoadjuvant chemotherapy. We performed immunohistochemical stainings of P53, P16, maspin, PTEN, BMI1 and Ki67, characterized the subcellular localization and related staining outcome with chemotherapy response and overall survival. Binary logistic regression analysis was used to analyze chemotherapy response and Kaplan-Meier-analysis as well as the Cox proportional hazards model was applied for analysis of patient survival.

**Results:**

No significant associations between biomarker expression in BXs and patient survival or chemotherapy response were detected. In univariate analysis, positive immunohistochemistry of P53 (*P* = 0.008) and P16 (P16; *P* = 0.033) in RXs was significantly associated with poor survival prognosis. In addition, presence of P16 in RXs was associated with poor survival in multivariate regression analysis (*P* = 0.003; *HR* = 0.067) while absence of P16 was associated with good chemotherapy response (*P* = 0.004; *OR* = 74.076). Presence of PTEN on tumor RXs was significantly associated with an improved survival prognosis (*P* = 0.022).

**Conclusions:**

Positive immunohistochemistry (IHC) of P16 and P53 in RXs was indicative for poor overall patient survival whereas positive IHC of PTEN was prognostic for good overall patient survival. In addition, we found that P16 might be a marker of osteosarcoma chemotherapy resistance. Therefore, our study supports the use of tumor RXs to assess the prognostic value of biomarkers.

**Electronic supplementary material:**

The online version of this article (doi:10.1186/s12885-015-1397-4) contains supplementary material, which is available to authorized users.

## Background

Osteosarcoma is the most common malignancy of bone and among the deadliest cancers in adolescents [1, 2]. Osteosarcoma patients are commonly treated with multiagent neoadjuvant chemotherapy, combined with surgery to remove the primary tumor mass and subsequent adjuvant chemotherapy. Introduction of chemotherapy has increased the mean 5-year survival rates of patients with localized disease from 20 % in the early 1970s to above 60 % at present [1, 3]. In contrast, the presence of metastases is a strong prognostic factor for poor survival rates of 30 % or less [4].

Specifically for osteosarcoma, a patient’s response to neoadjuvant chemotherapy has a considerable prognostic value and has therefore replaced single adjuvant chemotherapy [5, 6]. To date, necrosis of tumor resections (RXs) above 90 %, although only a crude read out, is still used in clinical practice due to its prognostic power for patient survival [4, 7]. Current protocols of neoadjuvant chemotherapy for routine use in osteosarcoma are based on combinations of highly cytotoxic drugs such as cisplatin, methotrexate and doxorubicin [8]. Although potent, these drugs are not specific enough and tumor resistance, subsequent disease progression as well as patient death are therefore frequently observed. Consequently, numerous immunohistochemical studies have tried to identify osteosarcoma-biomarkers. For instance, VEGF [9-11], ezrin [12-14], P53 [15], P16 [16], CD44 [17], CXCL12 [18] were evaluated as prognostic factors for survival, whereas immunohistochemical stainings of nuclear P16 [19], CRIP1 [20] and COX-2 [21] were investigated as predictors of chemotherapy response.

However, the above mentioned studies only evaluated chemotherapy naïve biopsies (BXs) of osteosarcomas. Despite the larger amounts of available tissue compared to needle biopsied tissue, fewer studies analyzed RXs because it is thought that no valid prediction about patient survival can be made. Nevertheless, analysis of marker expression in remaining viable tumor tissue after chemotherapy can be investigated similar to assessing the degree of response to chemotherapy [22], and may yield important information about patient prognosis and the impact of chemotherapy in non-responders. Similarly, only a few studies analyzed RXs of osteosarcomas in order to study expression changes of biomarkers prior to and after chemotherapy yet in these studies significant correlations were found between clinicopathological parameters and expression changes of biomarkers such as VEGF [23], MMP-2 [24], ezrin [25] and alkaline phosphatase [26].

Tumor suppressors are thought to have a major impact on the response to chemotherapy [27-30] and hence, patient survival. In this study, we therefore analyzed immunohistochemical stainings in BXs and RXs of four established tumor suppressors (P53, P16, PTEN and maspin) in viable patient-derived tissue before and after neoadjuvant chemotherapy in order to better understand their changes during chemotherapy, and to find out if this change is related to chemotherapy response or survival. Wild-type P53 is a major player in the DNA damage response and initiates cell apoptosis once the extent of DNA damage is beyond repair [31]. Intriguingly, wild-type P53 also has the ability to protect tumors during chemotherapy [27], highlighting the need for a better characterization of P53 as a marker during chemotherapy. P53 is well known to be mutated in high grade osteosarcomas [15, 32] and mutant P53 is often detected by immunohistochemistry (IHC) due to its increased half-life [33], highlighting its potential as a valuable marker for osteosarcoma patient prognosis.

P16 is considered another major tumor suppressor and acts through blocking of cyclin dependent kinase 4 signaling and consequently, cell cycle progression [34]. P16 as a biomarker is less well characterized than P53 in osteosarcoma. Nevertheless, the use of osteosarcoma BXs identified P16 as a sensitive prognostic factor [35] and to be predictive for good chemotherapy response [19]. In contrast to what is inferred from biopsied samples, changes in P16 might protect the tumor cells during chemotherapy by decreasing their proliferation rate [28]. Therefore, it is of importance to not only study the presence of individual tumor suppressors but to also investigate their impact on tumor proliferation (e.g., monitoring of Ki67 [36]). Tumor proliferation measured by Ki67 indices is believed to have strong prognostic value in multiple types of cancer [37-39]. Proliferation is the result of an excess of growth promoting signals such as growth-factor signaling pathways or the inhibition of cell cycle regulators. Thus, proliferation can be altered at various levels, for instance through upregulation of BMI1, causing a deactivation of P16 and hence, an increase in proliferation [40]. At the same time, BMI1 was found to be overexpressed in more than half of chemotherapy naïve osteosarcoma specimens [41]. To date, no significant correlation between clinicopathological parameters and BMI1 expression in osteosarcomas was discovered.

Two additional tumor suppressors, maspin and PTEN, have hardly been studied in the context of osteosarcoma so far. Similar to P16 [34], PTEN controls cell proliferation by regulating cyclin D levels and inhibiting PI3K-Akt signalling [42]. Thus, presence of PTEN in tumor specimens is considered as being prognostic for good patient survival [43, 44]. The precise mechanism of maspin, on the other hand, is still under debate. On the one hand, studies showed a reduced metastatic potential of breast cancer cells [45] or augmented cancer cell death by chemotherapeutic drugs through induction of maspin [29] and, on the other hand, studies demonstrated increased expression of maspin to be an indicator of poor survival [46] or poor chemotherapy response [47]. These controversial results suggest that expression changes of maspin are rather secondary effects caused by similar changes to adjacent and more relevant genes, further supported by a recent study [48].

Drugs used in current chemotherapeutic protocols to treat osteosarcomas generally induce tumor cell death, yet some tumors adapt in order to avoid death. In order to identify patients at risk, we studied the before mentioned biomarkers in primary osteosarcoma tissues before and after chemotherapy. Using immunohistochemistry (IHC), we evaluated associations between the presented biomarkers and clinical parameters such as overall survival, response to chemotherapy, metastasis or proliferation of the primary tumor in order to assess the clinical value of the studied biomarkers.

## Methods

### Patient samples

This retrospective study was conducted with tumor tissue specimens from a total of 61 patients who were operated between December 1987 and October 2005. The specimens were retrieved from the archive of the Institute of Surgical Pathology of the University Hospital, Zurich, Switzerland. All tissue samples were graded as high grade osteosarcomas according to the current histopathological classification by the World Health Organization [49]. Follow-up was started at first diagnosis of the osteosarcoma and ended at death or with the last clinical record in our hospital database giving a range of 7–210 months with a median follow-up of 85 (BX) and 90 (RX) months. All patients used for survival analysis had a complete clinical record and a follow-up of at least 50 months. Patients receiving complete neoadjuvant chemotherapy according to the formerly used COSS protocols, namely COSS-86, COSS-91 and COSS-96 [50, 51] were retrospectively selected and the corresponding clinical records were reviewed and updated. Tumor response was evaluated based on the grading of tumor necrosis according to the guidelines by Salzer-Kuntschik *et al.* [22]. Patients were termed “responders” if tumor necrosis, based on histopathological analysis, was greater than 90 % after neoadjuvant chemotherapy and “non-responders” if less than 90 % of the tumor was necrotic. Ultimately, panels of 47 chemotherapy naïve biopsies (herein termed BXs), 44 neoadjuvant chemotherapy treated tumor samples (i.e., resections, herein termed RXs) and 11 lung metastasis-derived tissues were analyzed. In a maximum of 31 cases, a matched chemotherapy-naïve BX and neoadjuvant chemotherapy treated RX of the same patient were obtained and used for the analysis of changes of IHC in BXs and RXs derived from the same patient.

### Tissue microarray

In this study a tissue microarray (TMA) containing paraffin-embedded primary tumor material (both BXs and RXs as well as lung metastases, see ref [52]) was used to assess marker expression. Based on hematoxylin and eosin stained sections of the tumor, viable tumor cell containing areas were selected for the construction of the TMA. All BX-derived available tissue cores with sufficient numbers of tumor cells were evaluated. For RX derived material, only tissue cores derived from “non-responders” (defined as Salzer-Kuntschik grade 4–6) and “responders” (Salzer-Kuntschik grade 2 and 3) were considered for analysis, due to a lack of viable tissue in grade 1 “complete responders”.

### Immunohistochemistry and TMA analysis

Immunohistochemistry (IHC) was carried out on 4 μm sections of the TMA. Sections were transferred to an adhesive-coated slide system (Instrumedics, Hackensack, NJ, USA), deparaffinized, and processed with an automated Ventana Benchmark staining system (Ventana Medical Systems Tucson, Arizona, USA). Heat-mediated antigen retrieval was performed with cell conditioner 1 for at least 30 min. Individual sections were probed with the following antibodies: mouse monoclonal anti-P16^ink4a^ (clone 16P04, dilution 1:600; LabVision/Neomarkers, USA), mouse monoclonal anti-P53 (clone DO-7, dilution 1:80; Dako, DNK), mouse monoclonal anti-PTEN (dilution 1:200; clone 28H6; Leica Biosystems/Novocastra, GER), mouse monoclonal anti-maspin (clone G167-70, dilution 1:200, BD Pharmingen, USA), mouse monoclonal anti-BMI1 (clone F6, dilution 1:50; Millipore/Upstate, USA) and mouse monoclonal anti-Ki67 (clone MIB-1, dilution 1:20; Dako, DNK). Visualization of the antibody binding was done by applying the iVIEW DAB Kit (Ventana Medical Systems Tucson, Arizona, USA). Slides were counterstained with hematoxylin. A pathologist (CP) and an instructed scientist (BR) independently analyzed the tissue cores in a blinded fashion, where special attention was given to the subcellular (nuclear or cytoplasmic) localization of the analyzed marker. A consensus grading was formed in case of differences between individual samples. At least two cores per patient sample were analyzed to compensate for tissue heterogeneity. Tissue cores were graded as “negative” (grade 0) if less than 10 % of the tumor cells were stained, as “positive” (grade 1) if between 10 and 50 % of the tumor cells were immunostained with intermediate or high intensity and as “strongly positive” (grade 2) if more than 50 % of the tumor cells were stained with high intensity. In addition, changes of biomarkers following chemotherapy were investigated by comparing the immunohistochemical grades of BXs and RXs of the same patient. These changes were classified as “increase”, “no change” or “decrease” of the respective biomarker.

### Statistical analysis

Kaplan-Meier curves were used to calculate overall patient survival, which was defined as the time from diagnosis until death or until last follow-up. Log-rank tests were used to assess the statistical difference between groups. Multivariate Cox regression models were used to calculate hazard ratios (HRs) and 95 % confidence intervals (CIs). The clinicopathologic factors patient age, gender, location of primary tumor occurrence and histological subtype of osteosarcoma were included as covariates next to expression of individual biomarkers. Multivariate binary logistic regression models were used to estimate Odds ratios (ORs) as well as 95 % CIs. To determine associations between biomarker expression and other parameters (i.e., proliferation (Ki67 immunostaining); presence of metastasis) Fisher’s exact tests were applied. All statistical tests were 2-sided where *P* < 0.05 was regarded as statistically significant. PASW Statistics 18.0 (IBM Corp., USA) was used for statistical evaluation.

### Ethics statement

The design of this retrospective study was assessed and approved by the local ethics committee of the University Hospital Zurich (approval reference number StV 41–2005).

## Results

### Patient cohort characteristics

As depicted in Table 1, the two patient cohorts used for analyses of BXs or RXs had similar clinicopathological characteristics. In both cohorts, the majority of the patients were male (BX: 60 %, RX: 64 %) and the overall mean age was 18.4 years and 18.0 years in the BX cohort and RX cohort, respectively. Most osteosarcomas were seen in patients aged 10–24 years (BX: 65 %, RX: 66 %). The distribution of histological subtypes such as the predominant osteoblastic type (BX: 71 %, RX: 70 %) or the main sites of primary tumor occurrence such as the tibia/ fibula/ calcaneus (BX: 40 %, RX: 32 %) or the femur (BX: 38, RX: 43 %) were similar in both patient cohorts. A total of 65 % (BX) and 68 % (RX) of patients were alive at the last follow-up resulting in similar five-year survival rates of 65 % (BX) and 68 % (RX). Chemotherapy response (≥90 % tumor necrosis) subsequent to neoadjuvant chemotherapy was found in 54 % (BX) and 49 % (RX) of the patients compared to 46 % (BX) and 51 % (RX) being non-responders (<90 % tumor necrosis). During follow-up, 44 % (BX) and 38 % (RX) of patients developed metastases.

**Table 1:** Clinicopathologic characteristics of high-grade osteosarcoma patients and IHC of six biomarkers

Variables	n_BX_	%_BX_	n_RX_	%_RX_
All high grade osteosarcoma	48	100	47	100
Neoadjuvant chemotherapy	48	100	47	100
Sex				
Female	19	40	17	36
Male	29	60	30	64
Patient age				
<10 years	10	21	9	19
10–24 years	31	65	31	66
>24 years	7	15	7	15
Histological subtype				
Osteoblastic	34	71	33	70
Chondroblastic	4	8	7	15
Fibroblastic	5	10	4	9
Telangiectatic	5	10	3	6
Location				
Tibia / Fibula / Calcaneus	19	40	15	32
Femur	18	38	20	43
Humerus / Ulna	6	13	5	11
Axial	5	10	7	15
Pathologic Response				
Responder	26	54	24	49
Non-Responder	22	46	23	51
Metastasis				
Yes	21	44	18	38
No	27	56	29	62
P16 total (*n*_*matchedBX-RX*_ = 27)	44	100	39	100
P16_positive_	25	57	17	44
P16_negative_	19	43	22	56
P53 total (*n*_*matchedBX-RX*_ = 31)	47	100	44	100
P53_positive_	9	19	12	27
P53_negative_	38	81	32	73
PTEN total (*n*_*matchedBX-RX*_ = 10)	40	100	22	100
PTEN_positive_	25	63	7	32
PTEN_negative_	15	37	15	68
Maspin total (*n*_*matchedBX-RX*_ = 21)	39	100	33	100
Maspin_positive_	26	67	10	30
Maspin_negative_	13	33	23	70
Ki67 total (*n*_*matchedBX-RX*_ = 15)	43	100	25	100
Ki67_positive_	24	56	8	32
Ki67_negative_	19	44	17	68
BMI1 total (*n*_*matchedBX-RX*_ = 16)	42	100	28	100
BMI1_positive_	11	26	2	7
BMI1_negative_	31	74	26	93

*BX* biopsy, *RX* resection

### Tumor IHC

**Fig. 1:**
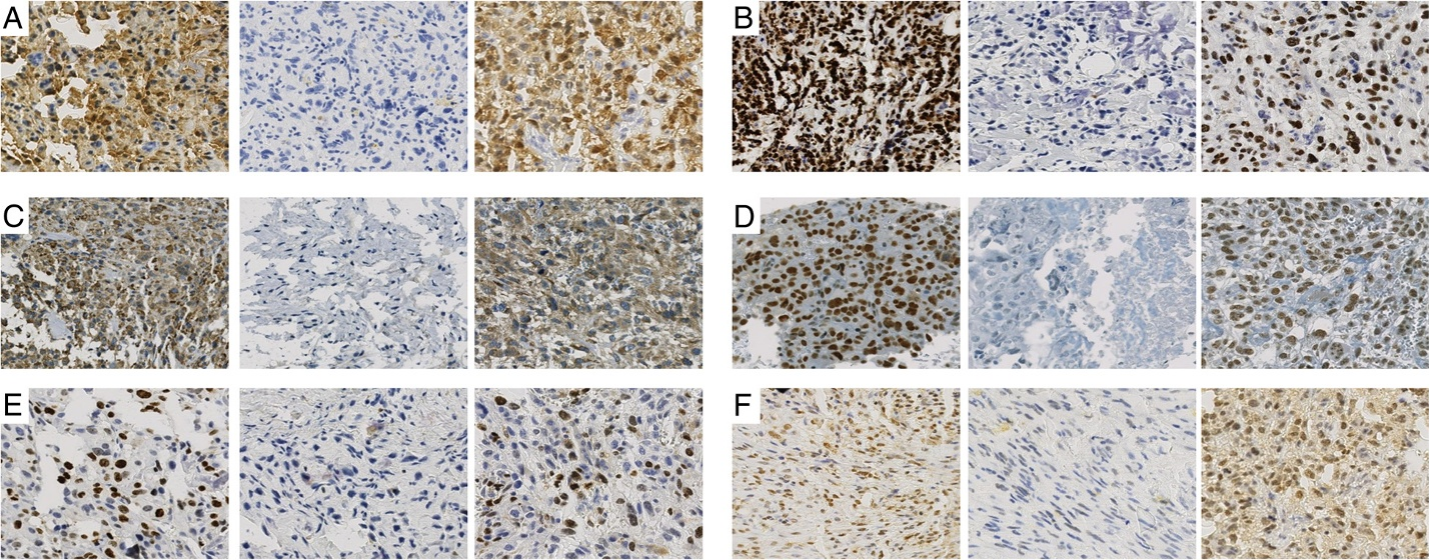
Representative images of immunohistochemistry of the six analyzed biomarkers. For each part (**a–f**) the same order of samples is shown: left (positive staining), middle (negative staining), right (positive staining of a lung metastasis). **a**, nuclear and cytoplasmic P16 staining. **b**, nuclear P53. **c**, cytoplasmic PTEN. **d**, nuclear maspin. **e**, nuclear Ki67. **f**, nuclear BMI1. Normalized magnification of all images, 40x; Hematoxylin counterstaining

Representative examples of positive as well as negative immunohistochemical stainings of BXs are given in Fig. 1. The subcellular localization differed between the analyzed markers: IHC of P53 (Fig. 1b), Maspin (Fig. 1d), Ki67 (Fig. 1e) and BMI1 (Fig. 1f) showed nuclear localization in >90 % of the positively stained BXs. In contrast, PTEN (Fig. 1c) was exclusively found in the cytoplasm. Subcellular localization of P53, Maspin, Ki67, BMI1 and PTEN in RXs was the same as in BXs. IHC of P16 (Fig. 1a) showed equal numbers of “cytoplasmic and nuclear” as well as “cytoplasmic only” (see Additional file 1) P16-positive BXs (52 % versus 48 % of the BX samples, respectively). In P16-positive RXs, “cytoplasmic only” expression of P16 was more frequent than “cytoplasmic and nuclear” localization of P16 (65 % versus 35 % of the RX samples, respectively). Furthermore, all P16-positive osteosarcoma samples had detectable P16 in the cytoplasm of cancer cells, whereas no sample was found with a “nuclear only” subcellular localization of P16.

Table 1 also indicates the numbers of samples available for each analyzed marker. In general, a larger number of BXs (n_BX_: 39–47) was available for IHC compared to specimens derived from RXs (n_RX_: 22–44). A positive BX staining was most often found for maspin and PTEN, in with approximately two thirds of the samples were positively stained. Staining of P53 and BMI1 was less common, with 19 % and 26 % positive staining, respectively. In over half of the BXs, Ki67 (56 %) and P16 (57 %) could be detected. As depicted in Table 1, chemotherapy decreased the immunohistochemical grade of P16, PTEN, maspin, Ki67 and BMI1, i.e., led to a decreased expression of the marker in the patient samples after chemotherapy. This decrease was most dramatic for BMI1 and maspin, where in relative terms, more than half of the samples lost their marker expression. In contrast, a relative increase of P53-positive samples was observed after chemotherapy (BX, positive: 19 %; RX, positive: 27 %).

### Survival analysis

**Fig. 2:**
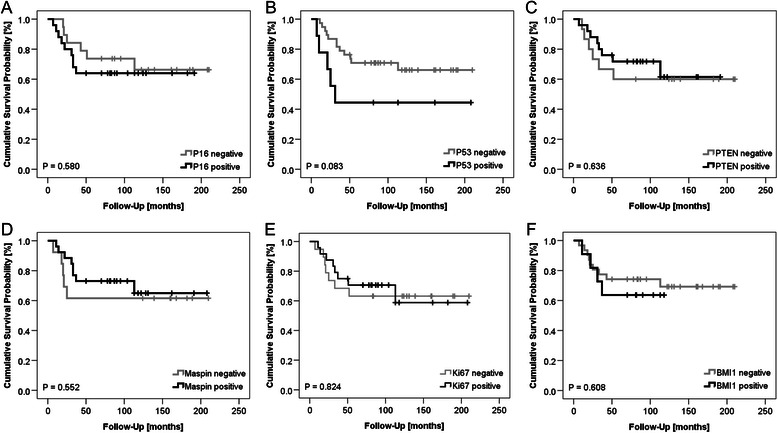
Univariate Kaplan-Meier survival analysis of biomarkers in BXs. Kaplan-Meier survival curves showing survival probabilities of patients according to their (**a**) P16, (**b**) P53, (**c**) PTEN, (**d**)x maspin, (**e**) Ki67 and (**f**) BMI1 expression status

**Fig. 3:**
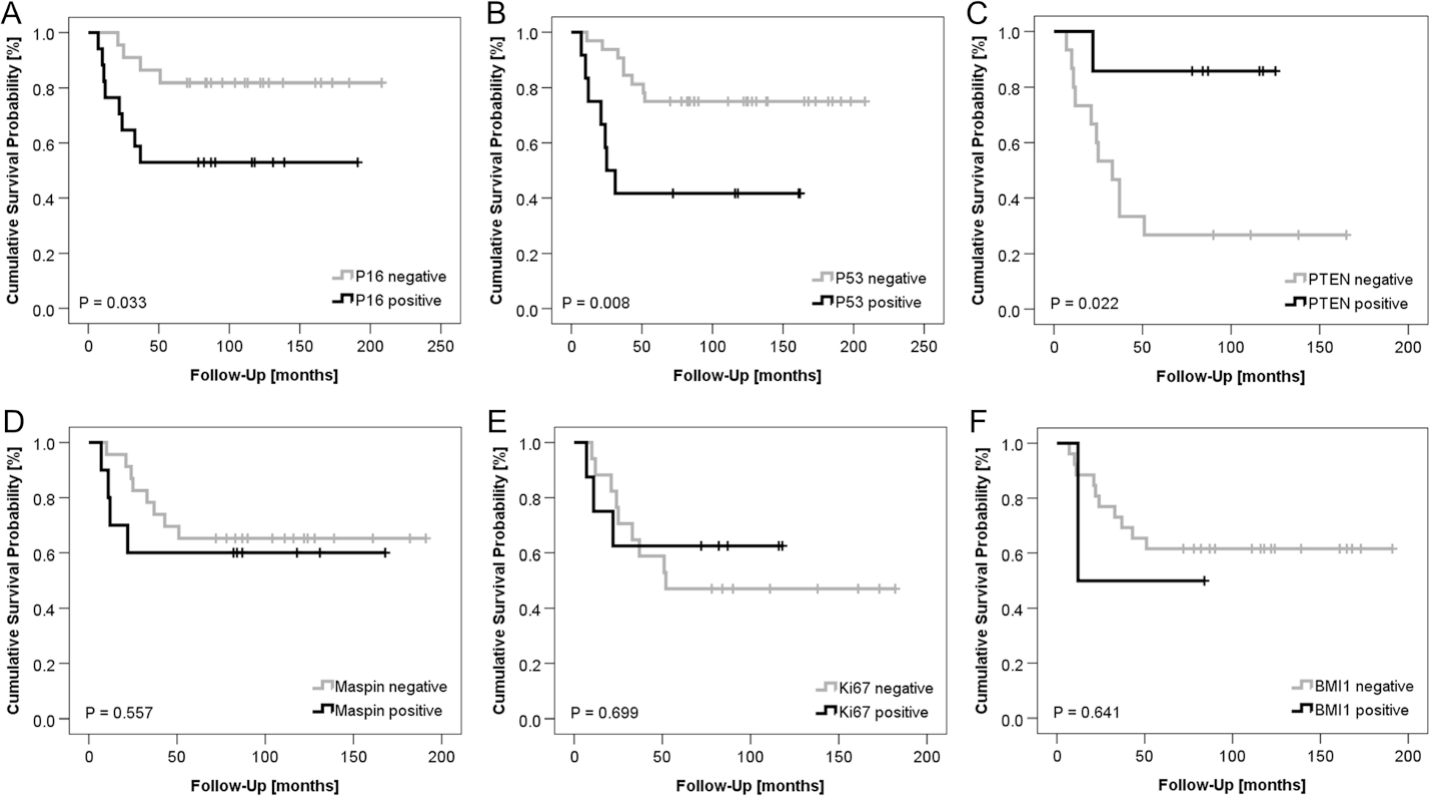
Univariate Kaplan-Meier survival analysis of biomarkers in RXs. Kaplan-Meier survival curves showing survival probabilities of patients according to their (**a**) P16, (**b**) P53, (**c**) PTEN, (**d**) maspin, (**e**) Ki67 and (**f**) BMI1 – expression status

As depicted in Fig. 2. Kaplan-Meier survival analysis of chemotherapy-naïve BXs of high grade osteosarcoma patients yielded no significant differences in overall survival for the various biomarkers, although for P53 a trend was observed for worse survival in case of presence of nuclear P53 (*P* = 0.083; Fig. 2b). In contrast, the analysis of patient RXs yielded significant differences in overall survival as illustrated in Fig. 3. Positive expression of P16 (*P* = 0.033; Fig. 3a) and P53 expression (*P* = 0.008; Fig. 3b) were found to be prognostic markers for poor overall survival of patients. In contrast, absence of PTEN (*P* = 0.022; Fig. 3c) in patient RXs was significantly associated with worse overall survival. Expression of maspin (Fig. 3d), Ki67 (Fig. 3e) and BMI1 (Fig. 3f) in RXs was not significantly associated with overall survival prognosis. Due to the fact that P16 was present in the “cytoplasm only” or in the “cytoplasm and nucleus” of some samples, we sought to see if there is a difference in survival rates between these two subgroups. However, Kaplan-Meier survival analysis did not yield a difference in survival probability according to the subcellular localization of P16 (see Additional file 2).

Cox regression analysis (Table 2A) demonstrated that no significant contribution of any biomarker was detected in BXs (data not shown), but in RXs, absence of P16 expression (*P* = 0.003; *HR* = 0.067; 95 % CI: 0.011 - 0.397) was a significant favorable prognostic factor for overall survival. The other biomarkers were not found to be associated with overall survival (Table 2). Similarly, clinicopathologic parameters such as age, gender, tumor location or tumor subtype possessed no prognostic value for patient survival in multivariate analyses.

**Table 2:** Multivariate analysis of patients with osteosarcomas receiving neoadjuvant chemotherapy

	A. Cox regression analysis of association between clinicopathologic variables and overall survival				B. Binary logistic regression analysis of association between clinicopathologic variables and tumor-response status			
Variables^a^	P-value	HR	95 % CI		P-value	OR	95 % CI	
Age	0.973				0.126			
<10 years	0.829	0.000	0.000	∞	0.043	0.006	0.000	0.845
10–24 years	0.922	0.876	0.063	12.227	0.084	0.012	0.000	1.814
Gender	0.122	4.390	0.674	28.596	0.609	2.189	0.109	44.045
Location	0.972				0.601			
Tibia/ fibula/calcaneus	0.743	0.000	0.000	∞	0.999	6.119E9	0.000	∞
Femur	0.737	0.000	0.000	∞	0.999	4.232E9	0.000	∞
Humerus/ulna	0.719	0.000	0.000	∞	0.999	5.229E10	0.000	∞
Histological subtype	0.902				0.935			
Chondroblastic	0.986	0.139	0.000	∞	0.999	8.367E8	0.000	∞
Fibroblastic	0.922	22186	0.000	∞	0.999	0.000	0.000	∞
Osteoblastic	0.932	6308	0.000	∞	0.514	5.300	0.036	789.165
RX P16 negative (*n* = 39)	0.003	0.067	0.011	0.397	0.004	74.076	3.875	1415.946
RX P53 negative (*n* = 44)	0.315	0.515	0.141	1.879	0.159	4.786	0.541	42.368
RX PTEN negative (*n* = 22)	0.166	5.342	0.498	57.310	0.999	2.169E9	0.000	∞
RX Maspin negative (*n* = 33)	0.409	0.482	0.085	2.725	0.192	4.384	0.477	40.278
RX Ki67 negative (*n* = 25)	0.069	0.157	0.021	1.157	0.999	1.934E9	0.000	∞
RX BMI1 negative (*n* = 28)	0.353	3.624	0.239	54.951	1.000	0.000	0.000	∞

In the upper section, the multivariate analysis including P16 is shown. In the lower section, statistical parameters of biomarker expression of separate multivariate analyses (including the same clinicopathologic factors from the upper section) are shown

*RX* resection, *HR* hazard ratio, *OR* Odds ratio, ∞ infinity

^a^Coding of variables was a follows: age: 1 (<10 year), 2 (10–24 years) and 3 (>24 year). Gender: 1 (female) and 2 (male). Location: 1 (tibia/ fibula/ calcaneus), 2 (femur), 3 (humerus/ ulna) and 4 (axial sites). Histological subtype: 1 (chondroblastic) 2 (fibroblastic), 3 (osteoblastic) and 4 (telangiectatic)

### Chemotherapy response

Chemotherapy response of the tumor following chemotherapy has a strong influence on patient survival prognosis. Therefore we used binary logistic regression to analyze the expression of biomarkers on BXs in connection with gender, patient age, location of tumor and histological subtype to determine the predictive value on tumor response. Female gender was the only significant predictive factor for good chemotherapy response after neoadjuvant chemotherapy using multivariate analysis for models with BMI1, Ki67, PTEN, P16 and P53 (data not shown). In the multivariate model established for maspin, no such link between female gender and good chemotherapy response was detected.

In RXs, a correlation between tumor response after neoadjuvant chemotherapy and biomarker expression was detected. Expression of P16 in RXs was the only biomarker showing a significant correlation with poor tumor response after chemotherapy (absence of P16 in RXs: *P* = 0.004; *OR* = 74.076; 95 % CI:3.875-1415.946; Table 2B). None of the other biomarkers analyzed in RXs returned a significant correlation with tumor response.

### Proliferation

In order to see if a link exists between expression of the analyzed biomarkers and proliferation, we correlated expression of P53, P16, maspin, PTEN or BMI1 with Ki67 (see Additional file 3). We found maspin expression to be positively correlating with Ki67 expression in osteosarcoma tissues (*P*_*BX*_ <0.001, *P*_*RX*_ = 0.008; Fisher’s exact test). Similarly, PTEN expression positively correlated with Ki67 expression (*P*_*BX*_ = 0.018, *P*_*RX*_ = 0.046; Fisher’s exact test). Interestingly, IHC of P16 (*P*_*BX*_ = 0.050, *P*_*RX*_ = 0.086; Fisher’s exact test) showed a borderline correlation whereas P53 did not significantly correlate with Ki67 on osteosarcoma samples. Despite the low number of BMI1 positive RXs, positive IHC of BMI1 on BXs showed a significantly positive correlation with the tumor proliferation marker Ki67 (*P*_*BX*_ = 0.001, *P*_*RX*_ = 0.505; Fisher’s exact test).

### Metastasis

Compared to primary tumor material, a higher percentage of lung metastases showed expression of the analyzed markers (examples are shown in Fig. 1, right colums). More than 50 % of positively stained samples were found in 91 % (P16), 82 % (PTEN and maspin), 60 % (Ki67) and 55 % (BMI) of all available lung metastases. In contrast, positive P53 staining was only present in 18 % of all available lung metastases.

In both BXs and RXs, no statistically significant correlations between the expression of any of the investigated biomarkers and the development of metastases during follow-up were detected. Analyses of BXs and RXs stained for P16, P53, PTEN (BXs), Ki67, Maspin and BMI1 all yielded *P*-values > 0.35 (Fisher’s exact test) except for the analysis of PTEN in RXs, where presence of PTEN was indicative for suppression of metastases (*P* = 0.063, Fisher’s exact test).

### Changes of biomarkers

**Fig. 4:**
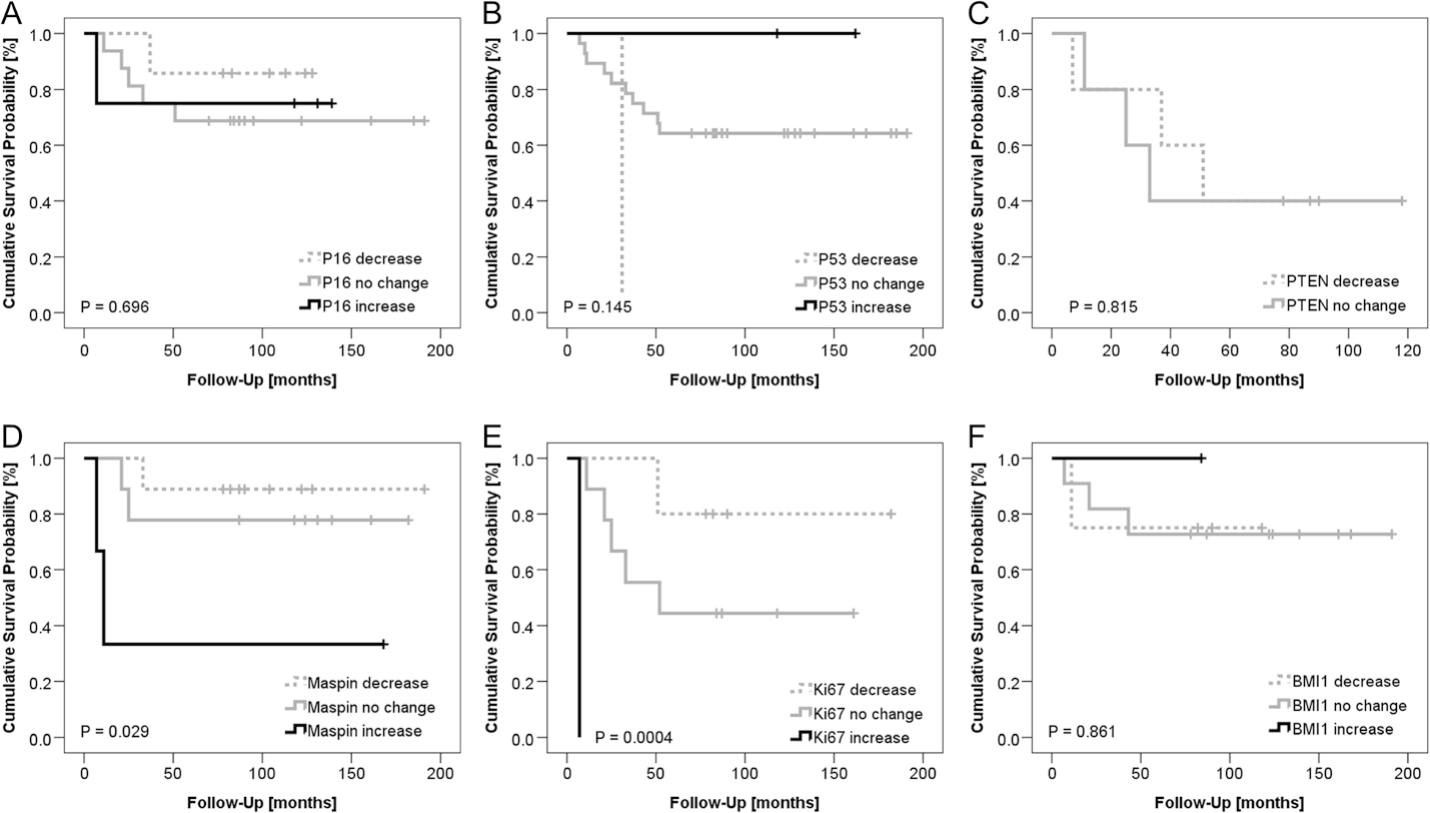
Univariate Kaplan-Meier survival analysis of biomarker expression changes prior to and after neoadjuvant chemotherapy in matched tissue samples. Kaplan-Meier survival curves showing survival probabilities of patients designated by the changes of IHC gradings of (**a**) P16, (**b**) P53, (**c**) PTEN, (**d**) maspin, (**e**) Ki67 and (**f**) BMI1

We investigated if changes in the histological grading of each marker (i.e., in BX and RX derived from the same patient) had a prognostic value for patient survival. Despite a limited number of matched patient tissues available (*n* = 10–31, exact sample numbers indicated in Table 1), we found that changes in gradings of Ki67 (*P* = 0.0004, log-rank test) and maspin (*P* = 0.029, log-rank test) had significant prognostic value for overall survival (Fig. 4), where a decrease in grading of both markers was associated with better survival compared to no change or an increase. Changes of biomarker gradings were neither significantly correlating with the formation of metastasis nor with chemotherapy response (data not shown).

## Discussion

We strongly believe that, especially in case of rare cancer entities such as osteosarcoma, all available tissue should be analyzed in order to gain more information about the molecular changes of the osteosarcoma during chemotherapy. By careful selection of the still viable resected material, valuable information can be obtained about patient survival prognosis or chemotherapy response. Here, we thus demonstrated prognostic roles of P53, P16 and PTEN in osteosarcoma by analyzing osteosarcoma samples after neoadjuvant chemotherapy. The prognostic value of P16 [53-55], P53 [15] as well as PTEN [43, 44] found in our study confirmed data found in other cancers. In addition to the common perception of analyzing chemotherapy naïve tissues in order to identify prognostic markers, we also demonstrated the value of analyzing (matching) resected tumor tissue.

To date, hardly any studies analyzing matched osteosarcoma patient samples prior to and after neoadjuvant chemotherapy exist. For the first time, we showed significant correlations between changes of maspin as well as Ki67 and osteosarcoma patient survival. In general, little is known about the role of maspin in cancer progression and it is questioned if maspin is playing a role in tumor development, in particular breast cancer, at all [48]. We are the first to study whether a metastasis-suppressing role of maspin exists in osteosarcoma, yet we could not detect a significant correlation between maspin expression in BXs or RXs and the development of metastases or any other clinicopathological parameter. However, we found that an increase in maspin expression in matched tumor specimens (prior to and after chemotherapy) had a worse survival prognosis compared to patients in which maspin gradings decreased or remained unchanged. Explanations for this finding might be either the formation of mutants of maspin [56] or the induction of maspin expression by chemotherapeutics [29] without a direct impact on tumor biology [48]. In contrast, IHC of the proliferation marker Ki67 is often discussed as a strong prognostic factor, for instance in Ewing’s sarcomas [57] or breast cancer samples [38]. Despite low patient numbers and although IHC of Ki67 was not significantly linked to patient survival in our and another osteosarcoma study [58], changes of Ki67 IHC scores had prognostic value for poor survival. Thus, one can speculate that osteosarcomas with increased proliferation rates might possess a survival advantage during cisplatin-based chemotherapy and ultimately lead to poorer patient survival. However, as the number of matched samples was limited, repetition of these analyses in a larger cohort should be performed.

With respect to P16, studies so far only focussed on the role of nuclear P16 in osteosarcoma. Positive expression of nuclear P16 was considered to be beneficial for overall survival rates of osteosarcoma patients [16] as well as predictive for good response after standard neoadjuvant chemotherapy [19]. These two studies investigated the role of nuclear P16 in chemotherapy naïve samples [16, 19] and showed that nuclear P16 suppressed the formation of osteosarcoma and increased the chances of success of neoadjuvant chemotherapy. Importantly, P16 in our study was predominantly present in the cytoplasm of positively stained samples, and hence, was related to a poor chemotherapy response and poor patient survival. This difference may be related by different functions of P16 in the nucleus and cytoplasm, or simply reflect the fact that cytoplasmic P16 is not available to exert its regulatory function inside the nucleus. In general, these findings are in line with studies describing other cancer types which claimed cytoplasmic P16 to be an indicator for advanced tumor stages [59] and increased aggressiveness of squamous cell carcinomas of the skin [60] or the cervix [61]. Analysis of BXs of head and neck tumors significantly linked different subcellular localizations of P16 to differences in patient survival, where strong cytoplasmic P16 was found to be prognostic for poor survival [55].

Our findings confirm findings from previous studies reporting P53 (in BXs) as a marker for osteosarcoma patient survival prognosis [15]. However, using IHC, only mutant P53 can be detected due to its prolonged half-life [62-64]. Mutant P53 is often found to be incapable of inducing DNA-damage signaling and thus, renders tumor cells apoptosis resistant. An increase in mutations of P53 might be a consequence of cisplatin-based chemotherapy [65] and proteins such as maspin might be prone to similar events and are therefore found overexpressed in osteosarcoma samples. In contrast, the P16-coding CDKN2A locus was shown to be deleted rather than mutated in osteosarcoma samples leading to a loss of P16 expression [66]. However, these results were derived from samples which were chemotherapy naïve and sequencing approaches of P16 positive RXs would be required to confirm the absence of mutations in the CDKN2A locus.

The tumor suppressor PTEN is frequently deactivated through deletions, leading to low or no PTEN expression in osteosarcoma samples [67]. Consequently, loss of PTEN leads to a more malignant phenotype and a poor patient survival. We demonstrated for the first time a better prognosis of osteosarcoma patients if their RXs were positively stained for PTEN. In line with our results, other studies also showed worse patient survival in case PTEN was absent [43, 44, 68].

Patients who present with metastatic disease have generally lower survival rates than patients with localized osteosarcomas [3]. In order to identify potential markers of metastasis, we correlated the studied biomarkers with the presence of metastases. Based on our results, none of the studied biomarkers showed any correlation with the development of metastases, irrespective of the origin of the evaluated tissue (e.g., BXs or RXs). These results point at a minor role of the studied biomarkers in the process of metastasis in osteosarcoma, although markers like P53, P16 or PTEN have a significant correlation with the overall survival of the patients included in this cohort study.

It is commonly known that males are more often affected by osteosarcoma than females [4], yet no difference in terms of response to chemotherapy has so far been shown between genders. Our analysis of osteosarcoma BXs showed a better response of female osteosarcoma-bearing patients upon neoadjuvant chemotherapy. Although the cause of this gender difference is unknown, it is unlikely to be an artefact as this outcome is in line with the generally higher survival rates of female osteosarcoma patients compared to male patients [69-71].

There are some limitations to our study. First, it is limited by low sample numbers, especially with respect to analysis of matched samples. Nevertheless, in comparison to other studies investigating biomarkers of osteosarcoma, our patient cohort can be considered as average sized [15]. Furthermore, additional analyses such as mutational analyses were not performed at this stage and although IHC is a commonly used method in the clinics it does not provide complete information about the functional state of the detected proteins. Nevertheless, IHC is a commonly available tool to identify the expression status of a protein in various tissues and to learn about the subcellular distribution of a protein within cancer cells.

## Conclusions

In conclusion, using resected material, we have identified P16 and PTEN as prognostic markers for poor and for good overall survival of osteosarcoma patients, respectively. We also showed potential evidence of P16 in causing poor chemotherapy response upon neoadjuvant chemotherapy, thus establishing a basis for future research on the role of P16 in chemotherapy resistance. Importantly, the use of matched BXs and RXs also allowed us to gain more insight in the dynamics of biomarker expression following chemotherapy. Ultimately our study demonstrates that the use of RXs yields many clues regarding chemotherapy response and patient survival, and thus, should be considered in addition to immunohistochemical evaluation of chemotherapy naïve material.

## Additional files

Additional file 1: **Positive, cytoplasmic only P16 immunostaining.** This figure displays an immunostaining of P16 which is solely present in the cytoplasm of tumor cells (arrows point at representative cancer cells). The blue color of all nuclei is never fully covered with brown DAB reagent, showing the extranuclear localization of the P16 staining.
Additional file 2: **Kaplan- Meier survival analysis of samples grouped according to subcellular localization of P16.** P16 positive samples were grouped according to the subcellular localization of P16, yielding “cytoplasmic and nuclear” (cn) P16 as well as “cytoplasmic only” (c) P16. (A) Kaplan-Meier plot showing no difference in survival rates of BXs stratified according to c or cn subcellular localizations of P16 (*P* = 0.358). (B) Kaplan-Meier survival analysis using RXs yielded similar survival rates of cn and c P16 (*P* = 0.845). In contrast, cnP16 and cP16 showed worse survival rates when compared to P16 negative samples (*P* = 0.067 and *P* = 0.059, respectively). Abbreviations: BX, biopsy; c, cytoplasmic only; cn, cytoplasmic and nuclear; RX, resection.
Additional file 3: **Correlations between Ki67 IHC and IHC of other biomarkers.** In general, Ki67 is used as a marker for proliferation. To see if the studied putatitve tumor suppressors and BMI1 can be correlated to the proliferation state of the analyzed osteosarcoma samples, Fisher’s exact tests were performed to evaluate significant correlations.
