# Supplementary material for: Prognostic value of tumor suppressors in osteosarcoma before and after neoadjuvant chemotherapy
Source: BMC Cancer. 2015 May 9;15:379. doi: 10.1186/s12885-015-1397-4 (PMC4435808; doi:10.1186/s12885-015-1397-4)
Supplement: Additional file 1: — Positive, cytoplasmic only P16 immunostaining. This figure displays an immunostaining of P16 which is solely present in the cytoplasm of tumor cells (arrows point at representative cancer cells). The blue color of all nuclei is never fully covered with brown DAB reagent, showing the extranuclear localization of the P16 staining. [file 12885_2015_1397_MOESM1_ESM.pdf]

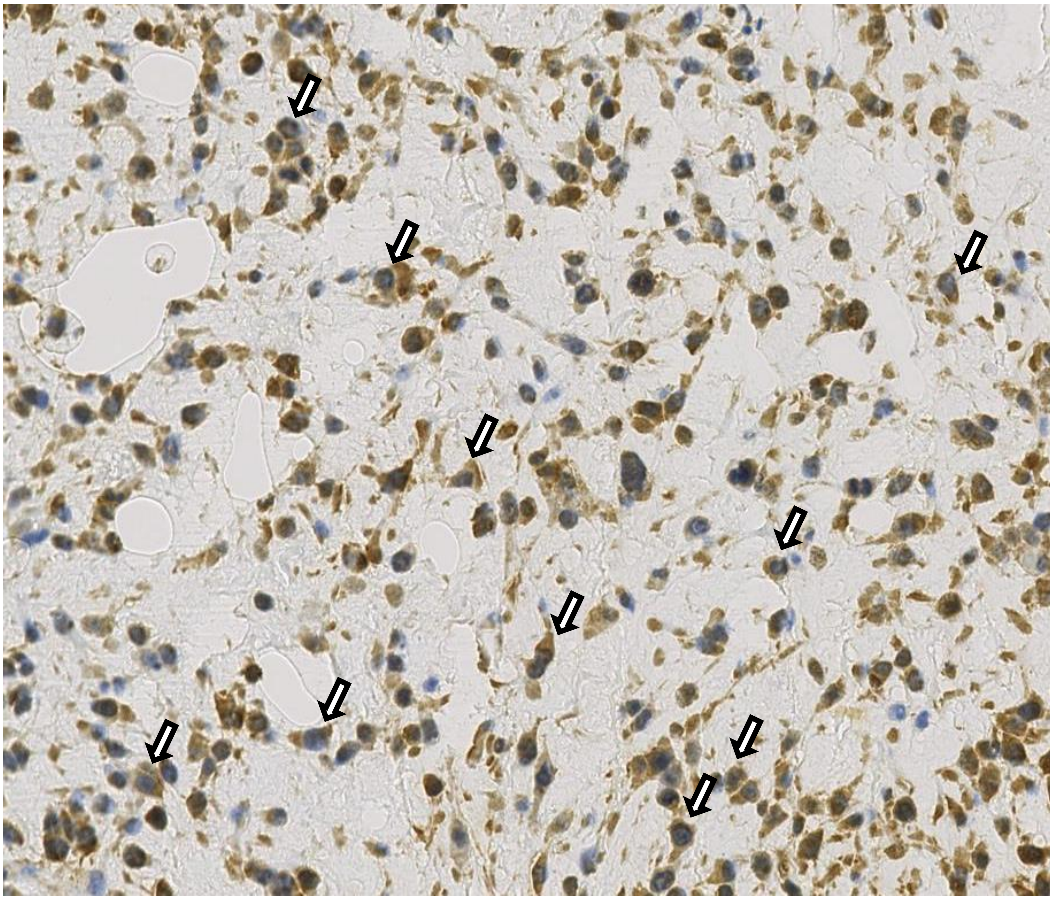

Additional file 1. Positive, cytoplasmic only P16 immunostaining. This figure displays an immunostaining of P16 which is solely present in the cytoplasm of tumor cells (arrows point at representative cancer cells). The blue color of all nuclei is never fully covered with brown DAB reagent, showing the extranuclear localization of the P16 staining.
