# Supplementary material for: Prognostic value of tumor suppressors in osteosarcoma before and after neoadjuvant chemotherapy
Source: BMC Cancer. 2015 May 9;15:379. doi: 10.1186/s12885-015-1397-4 (PMC4435808; doi:10.1186/s12885-015-1397-4)
Supplement: Additional file 2: — Kaplan- Meier survival analysis of samples grouped according to subcellular localization of P16. P16 positive samples were grouped according to the subcellular localization of P16, yielding “cytoplasmic and nuclear” (cn) P16 as well as “cytoplasmic only” (c) P16. (A) Kaplan-Meier plot showing no difference in survival rates of BXs stratified according to c or cn subcellular localizations of P16 (P = 0.358). (B) Kaplan-Meier survival analysis using RXs yielded similar survival rates of cn and c P16 (P = 0.845). In contrast, cnP16 and cP16 showed worse survival rates when compared to P16 negative samples (P = 0.067 and P = 0.059, respectively). Abbreviations: BX, biopsy; c, cytoplasmic only; cn, cytoplasmic and nuclear; RX, resection. [file 12885_2015_1397_MOESM2_ESM.pdf]

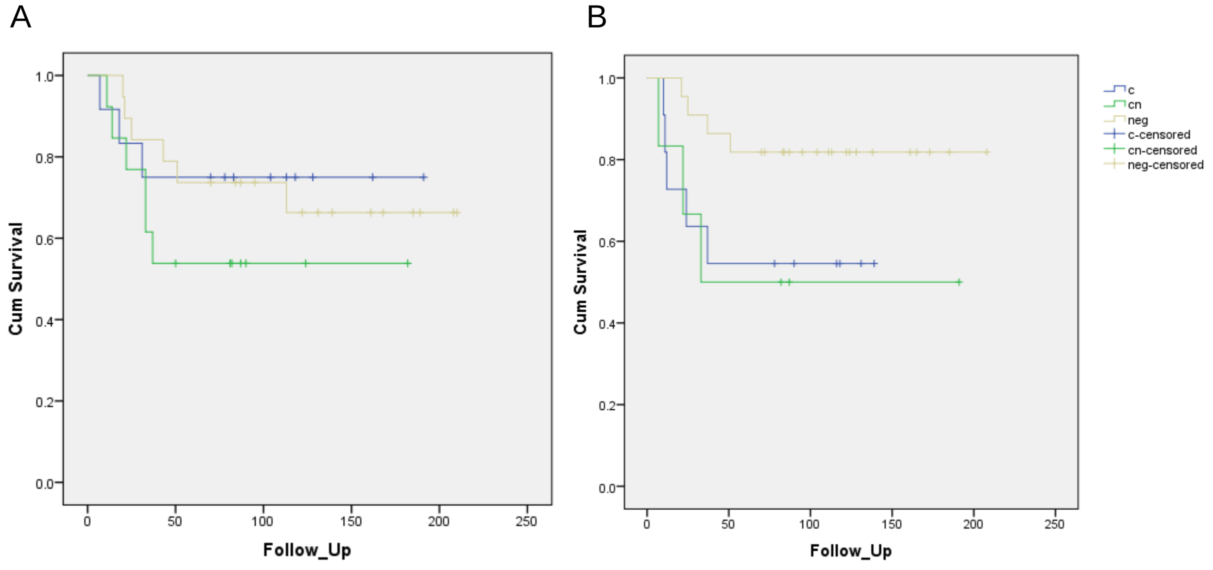

Additional file 2. Kaplan- Meier survival analysis of samples grouped according to subcellular localization of P16. P16 positive samples were grouped according to the subcellular localization of P16, yielding "cytoplasmic and nuclear" (cn) P16 as well as "cytoplasmic only" (c) P16. **(A)** Kaplan-Meier plot showing no difference in survival rates of BXs stratified according to c or cn subcellular localizations of P16 (P = 0.358). **(B)** Kaplan-Meier survival analysis using RXs yielded similar survival rates of cn and c P16 (P = 0.845). In contrast, cnP16 and cP16 showed worse survival rates when compared to P16 negative samples (P = 0.067 and P = 0.059, respectively). Abbreviations: BX, biopsy; c, cytoplasmic only; cn, cytoplasmic and nuclear; RX, resection;
