# Supplementary material for: Prognostic value of tumor suppressors in osteosarcoma before and after neoadjuvant chemotherapy
Source: BMC Cancer. 2015 May 9;15:379. doi: 10.1186/s12885-015-1397-4 (PMC4435808; doi:10.1186/s12885-015-1397-4)
Supplement: Additional file 3: — Correlations between Ki67 IHC and IHC of other biomarkers. In general, Ki67 is used as a marker for proliferation. To see if the studied putatitve tumor suppressors and BMI1 can be correlated to the proliferation state of the analyzed osteosarcoma samples, Fisher’s exact tests were performed to evaluate significant correlations. [file 12885_2015_1397_MOESM3_ESM.pdf]

Additional file 3. Correlations between Ki67 IHC and IHC of other biomarkers

		BX				RX			
		Maspin	neg	pos		Maspin	neg	pos	
Ki67	neg		11	5	P < 0.001		12	2	P = 0.008
	pos		1	21			2	6	
		PTEN	neg	pos		PTEN	neg	pos	
Ki67	neg		10	6	P = 0.018		11	2	P = 0.046
	pos		5	19			2	4	
		P53	neg	pos		P53	neg	pos	
Ki67	neg		15	4	P = 1.000		11	6	P = 0.667
	pos		20	4			4	4	
		P16	neg	pos		P16	neg	pos	
Ki67	neg		10	7	P = 0.050		8	7	P = 0.086
	pos		6	18			1	7	
		BMI1	neg	pos		BMI1	neg	pos	
Ki67	neg		17	0	P = 0.001		11	2	P = 0.505
	pos		12	11			8	0	

Abbreviations: IHC, immunohistochemistry; neg, negative; pos, positive.
